# Reflux Extension Index as a novel ordinal tool for quantifying saphenous reflux burden in women with Clinical-Etiological-Anatomical-Pathophysiological classes C1 to C2 chronic venous disease

**DOI:** 10.1016/j.jvsv.2026.102640

**Published:** 2026-10-10

**Authors:** Claudia Carvalho Sathler de Melo, Fausto Miranda Júnior, Adriana Sanudo, Ronald Luiz Gomes Flumignan

**Affiliations:** a Vascular and Endovascular Surgery Division, Department of Surgery, Universidade Federal de São Paulo (UNIFESP), São Paulo, Brazil; b ANGIOVASC Clinic, Nova Lima, Minas Gerais, Brazil

**Keywords:** Venous insufficiency, Saphenous vein, Duplex ultrasound study, Severity of illness index, women

## Abstract

**Objective:**

The study aimed to describe and quantify saphenous reflux patterns in women with early chronic venous disease (Clinical-Etiological-Anatomical-Pathophysiological classes C1 and C2), evaluate the ability of these patterns to discriminate between C1 and C2, and identify whether a greater extent of saphenous reflux corresponds to a greater probability of C2 classification.

**Methods:**

Cross-sectional study of women ≥18 years classified as Clinical-Etiological-Anatomical-Pathophysiological classes C1 or C2 by clinical examination and duplex ultrasound study between January 2019 and July 2023 at a private phlebology clinic; met inclusion criteria 1138 (2276 limbs); all examinations were performed by a single operator. The number of zones with reflux (0-12, Pittaluga lower-limb mapping) was binned into Reflux Extension Index (REXI-0; 0), REXI-1 (1-2), REXI-2 (3-5), and REXI-3 (≥6). Analyses were conducted at the limb level (n = 2276) and at the patient level for concordant subgroups (bilateral: C1, n = 666; C2, n = 240; total: n = 906). Logistic regression, adjusted for age and family history, was performed in 885 women with complete data. Model discrimination was assessed using the area under the receiver-operating characteristic curve (AUROC), monotonic trend within a generalized estimating equation framework, and McNemar test.

**Results:**

At the limb level, C2 prevalence increased monotonically with REXI category. At the patient level, adjusted odds ratios for bilateral C2 vs REXI-0 were 5.26 [95% confidence interval (CI), 3.34-8.29], 14.93 (95% CI, 9.31-23.95), and 51.97 (95% CI, 25.53-105.81) for REXI-1 to REXI-3 (all *P* < .001; McFadden pseudo-R^2^ = 0.280). Discrimination was good and improved with adjustment: crude AUROC 0.815 (95% CI, 0.783-0.847), adjusted 0.837 (95% CI, 0.805-0.868); and ΔAUROC, +0.022; *P* = .001. The adjusted probability of C2 crossed 50% between REXI-1 and REXI-2 (56.6%; 95% CI, 48.3-64.9). A left-sided C2 predominance was observed (McNemar odds ratio, 1.34; 95% CI, 1.04-1.74; *P* = .026). Of 240 women with bilateral C2, 101 (42.1%) had a REXI-0/REXI-1 profile—minimal or no saphenous reflux.

**Conclusions:**

REXI is a simple, quantitative, and clinically applicable ordinal framework for measuring saphenous reflux extension in women with early chronic venous disease. Prospective multicenter validation and interventional outcome studies are the necessary next steps.

**Article Highlights**

• **Type of Research:** A cross-sectional observational study of 2276 lower extremities in 1138 women with Clinical-Etiological-Anatomical-Pathophysiological (CEAP) classes C1 or C2 chronic venous disease, recruited from a single phlebology clinic between 2019 and 2023
• **Key Findings:** The Reflux Extension Index (REXI), a four-category ordinal index from a 12-zone saphenous duplex protocol, showed a monotonic association with CEAP class (generalized estimating equation trend odds ratio, 3.59; *P* < .001) and discriminated bilateral C2 from bilateral C1 disease at the patient level (crude AUROC, 0.815; adjusted, 0.837; ΔAUROC, +0.022; *P* = .001). In total, 42.1% of bilateral C2 women carried a C1-like profile (REXI-0 or REXI-1).
• **Take Home Message:** REXI converts CEAP classification into a quantitative index with low or absent reflux that may warrant monitoring clinical progression and prospective re-evaluation in future studies. Prospective multicenter validation and interventional outcome studies are the necessary next steps.

Chronic venous disease (CVD) is among the most prevalent vascular conditions worldwide, with a particularly high burden in women.^1^ The Clinical-Etiological-Anatomical-Pathophysiological (CEAP) classification system remains the internationally accepted standard for staging CVD severity and was most recently revised in 2020.^2^

The earliest clinically visible classes—C1 (telangiectasias and reticular veins) and C2 (varicose veins)—account for the majority of patients seeking phlebological care, particularly in aesthetic and outpatient settings. Their typical treatments are sclerotherapy, transcutaneous laser, or hybrid laser-sclerotherapy approaches for C1^3^, ^4^ targeting small varicose vessels directly; saphenous ablation in patients with CEAP C2 disease could be performed in the presence of symptoms and truncal reflux.

The literature on saphenous reflux patterns in C1 and C2 disease is sparse, heterogeneous, and largely qualitative. Engelhorn et al^5^ found saphenous reflux in 46% of C1 limbs, showed that segmental great saphenous vein (GSV) reflux predominates early,^6^ and described six anatomical patterns of GSV and small saphenous vein (SSV) reflux on duplex-Doppler.^7^ Coelho et al^8^ proposed further qualitative classification schemes. Pittaluga et al^9^ showed that reflux pattern and extent influence treatment selection and proposed an anatomical classification to guide surgery, and a zonal division of the lower limb for treatment planning.^10^ Yılmaz et al^11^ described a four-type classification by GSV reflux extent that correlated with clinical severity. Tan et al,^12^ in a 2024 systematic review (18 studies; 11,177 participants), confirmed that reflux pattern predicts clinical class and identified the unmet need to quantify reflux degree within the superficial system. The junctional reflux on the venous severity score study (352 patients; 10 centers) found that saphenofemoral junction reflux did not predict the Venous Clinical Severity Score, undermining any single anatomical landmark as a treatment criterion.^13^

Duplex ultrasound study is indispensable for accurate CEAP classification. Clinical examination alone is insufficient—particularly in overweight or obese patients, in whom varicose veins may be concealed by subcutaneous tissue.

## Methods

### Study design and setting

This is a cross-sectional observational study conducted at a private outpatient phlebology clinic in Nova Lima, Brazil, between January 1, 2019, and July 31, 2023, approved by the Universidade Federal de São Paulo Research Ethics Committee (Approval No. 7.390.422), which waived individual informed consent because only anonymized secondary data were used. Reporting followed the Strengthening the Reporting of Observational Studies in Epidemiology guideline (completed checklist in the Supplementary Material, online only).

### Study population

Of 1720 consecutive women screened, 582 were excluded for not meeting eligibility criteria, yielding 1138 women (2276 limbs). Inclusion criteria were female sex, age ≥18 years, and CEAP class C1 or C2 disease in one or both limbs confirmed by duplex.

#### Inclusion criteria

• Female sex
• Age ≥18 years
• CEAP clinical class C1 or C2 in one or both lower limbs, reflux confirmed by duplex ultrasound study (not by clinical inspection alone)

#### Exclusion criteria

CEAP class C0, C3 or higher, previous saphenous intervention, history of venous thromboembolism, venous compressive syndromes, lymphedema of any etiology, pregnancy or the early postpartum period, incomplete duplex examination or missing records. Male sex.

### CEAP classification

All participants were classified according to the 2020 CEAP system.^2^ C1 was defined by telangiectasias and/or reticular veins without varicose veins, and C2 by varicose veins. Classification was confirmed on duplex findings—no patient was assigned a CEAP class on inspection alone, ensuring accuracy independent of body habitus.

### Duplex ultrasound examination

All examinations were performed by a single certified and experienced operator (C.C.S.M.) on a Philips ClearVue 550 (Philips Healthcare) with a 4 to 12 MHz linear transducer. Reflux was defined as retrograde flow exceeding 0.5 s in the superficial and 1.0 s in deep system.^14^ Both limbs were examined systematically—GSV and tributaries medially, anterior saphenous, SSV and tributaries posteriorly. Perforator reflux was recorded separately and excluded from the number of zones with reflux (NZR), which quantifies the saphenous territory only.^15^ All duplex findings were recorded contemporaneously, at the time of examination, using a standardized zone-mapping (vascular cartography) form.

### The 12-zone saphenous framework and the NZR

Adapting Pittaluga lower-limb mapping concept,^10^ the superficial saphenous system of each limb was divided into 12 predefined anatomical zones: eight along the GSV territory including GSV and anterior saphenous (G1-G8), from the inguinal crease to the inferior margin of the medial malleolus, and four along the SSV territory (S1-S4), from a transverse line 4 cm above the popliteal crease—a boundary chosen to accommodate the highly variable termination of the SSV^16^, ^17^—to the inferior margin of the lateral malleolus (Fig 1). Each zone was scored as a discrete binary unit, reflux-positive or reflux-negative. A zone was counted as positive whenever pathological reflux (retrograde flow > 0.5 second) was identified in any portion of the saphenous trunk within that zone, irrespective of whether reflux occupied the whole zone or only part of it. Thus, a refluxing segment spanning, for example, one and a half zones was scored as two positive zones, since partial involvement of a zone was always counted as one whole positive zone. A continuous refluxing segment crossing more than one zone incremented the count for every zone it touched; discontinuous refluxing segments were scored independently. The NZR was the sum of reflux-positive zones (range, 0-12/limb).

**Fig 1:**
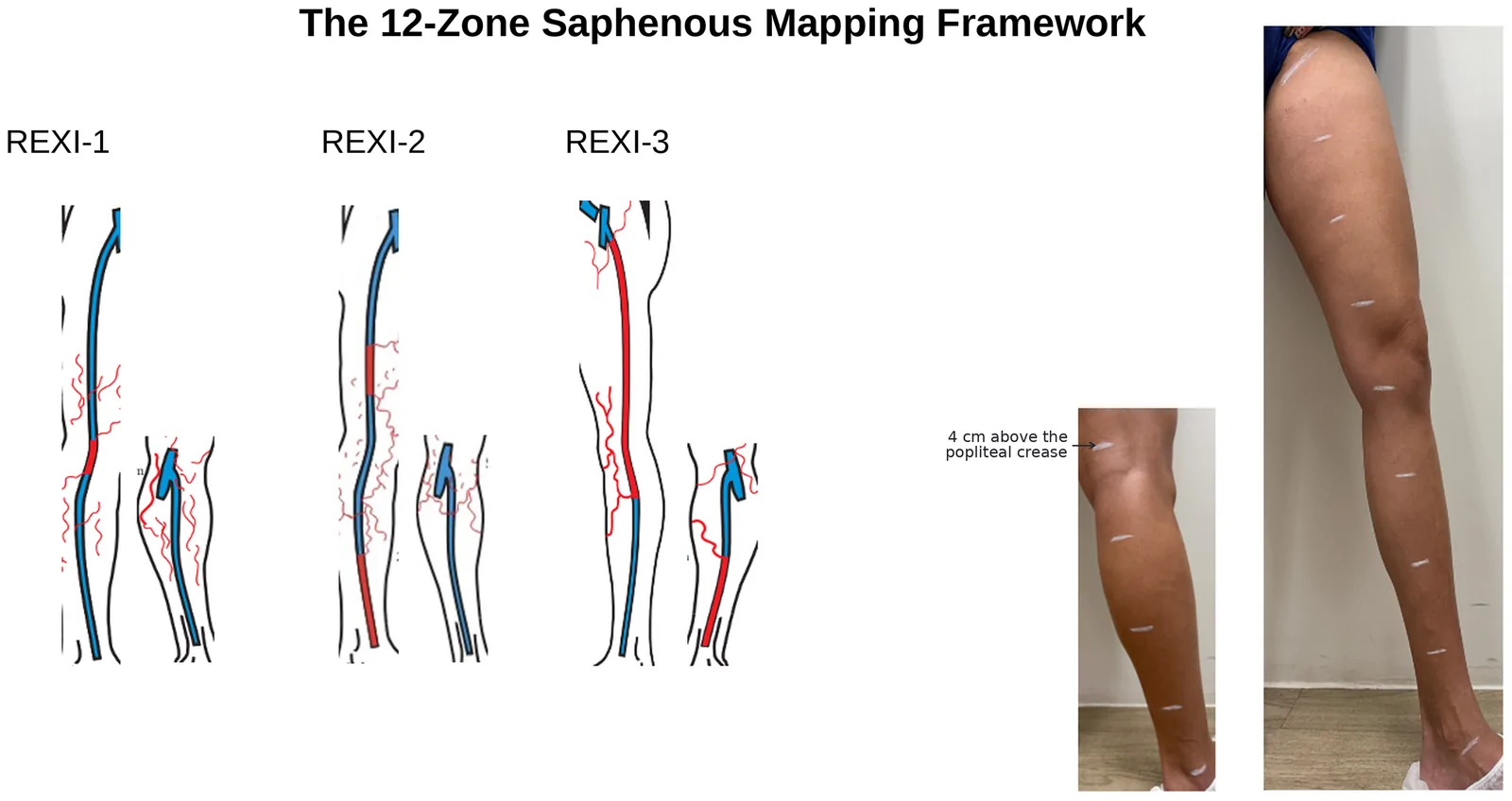
The 12-zone saphenous mapping framework. The superficial saphenous system of each limb is divided into eight great saphenous vein (GSV) zones (G1-G8) along the medial aspect and four small saphenous vein (SSV) zones (S1-S4) along the posterior leg. The three schematic panels illustrate representative Reflux Extension Index-1 (*REXI-1*) [number of zones with reflux (NZR) 1-2, minimal reflux], REXI-2 (NZR 3-5, moderate reflux), and REXI-3 (NZR ≥6, extensive reflux) patterns; *blue* denotes competent and *red* denotes refluxing saphenous segments and their associated reticular and telangiectatic networks. The clinical photographs demonstrate zone delineation on the medial (GSV) and posterior (SSV) aspects of the limb. The NZR is the per-limb count of saphenous zones with retrograde flow exceeding 0.5 second (range, 0-12).

Adapted from Pittaluga lower-limb division concept.^10^

### Reflux Extension Index

As this approach deliberately resolves a continuous—and frequently noninteger—reflux extent into whole-zone units, a degree of measurement granularity is inherent to the count. The Reflux Extension Index (REXI) was therefore defined as an ordinal four-category variable—REXI-0 (zone, 0), REXI-1 (zone, 1-2), REXI-2 (zone, 3-5), and REXI-3 (zone, ≥6)—specifically to absorb this within-zone variability and yield robust, clinically interpretable strata rather than a spuriously precise continuous score. REXI-0 served as the reference category in all comparative and regression analyses.

Fig 1 illustrates the 12-zone saphenous mapping framework, showing representative REXI-1 (NZR 1-2, minimal reflux), REXI-2 (NZR 3-5, moderate reflux), and REXI-3 (NZR ≥ 6, extensive reflux) patterns alongside clinical photographs demonstrating zone delineation for the GSV (8 zones) and SSV (4 zones).

### Concordant patient subgroups

Since each woman contributed two limbs that could differ in CEAP class, patient-level analyses were restricted to concordant subgroups—bilateral C1 (n = 666) and bilateral C2 (n = 240).

### Statistical analysis

Statistical methods are detailed in the Supplementary Material. In brief, continuous variables are reported as mean ± standard deviation, and categorical variables as frequencies and percentages. Analyses were conducted at the limb and patient levels, according to the unit of inference. Paired right-left differences in CEAP C2 classification were assessed using McNemar test, based on discordant pairs.

At the limb level, 2276 limbs from 1138 women were analyzed. Because each woman contributed two limbs, limb-level associations between REXI category and CEAP C2 classification were assessed using population-averaged logistic regression estimated via generalized estimating equations (GEEs), with an exchangeable working correlation structure and standard errors clustered at the patient level. REXI-0 was used as the reference category. Odds ratios (ORs) and 95% confidence intervals (CIs) were estimated for increasing REXI categories. Monotonic trend across ordered REXI categories was assessed within the GEE framework by modeling REXI as an ordinal variable.

At the patient level, analyses were restricted to women with concordant bilateral classification; women with discordant classification were described separately and excluded from regression. Crude and adjusted binary logistic regression models estimated the association between patient-level REXI category and bilateral C2 classification; adjusted models included age and family history of varicose veins. Model discrimination was assessed using the area under the receiver-operating characteristic curve (AUROC), with AUROCs compared using DeLong test. Adjusted predicted probabilities across REXI categories were estimated using marginal effects. All statistical tests were two-sided (α = .05). Statistical analysis was performed using STATA/MP version 18.0 (StataCorp LLC) and R version 4.3 (R Foundation for Statistical Computing; see Supplementary Material, online only).

## Results

### Sample characteristics

Of 1720 women screened, 582 were excluded, leaving 1138 women contributing 2276 limbs. The mean age of 44.1 years (standard deviation, 13.6; range, 18-83), and 67.5% of women were younger than 50 years of age. Venous symptoms were reported by 847 women (74.4%) and a family history of varicose veins by 904 of 1105 women with available data (81.8%). Sample characteristics are summarized in Table I.

**Table I:** Sample characteristics (n = 1138 women)

Characteristics	n (%)
Age, years (mean ± SD)	44.1 ± 13.6
Age range, years	18-83
18-29	150 (13.2)
30-39	337 (29.6)
40-49	281 (24.7)
50-59	197 (17.3)
≥60	173 (15.2)
Venous symptoms present	847 (74.4)
Family history present (n = 1105)	904 (81.8)
Limb-level CEAP—C1 limbs	1564 (68.7)
Limb-level CEAP—C2 limbs	712 (31.3)
Patient-level CEAP—bilateral C1	666 (58.5)
Patient-level CEAP—bilateral C2	240 (21.1)
Discordant right-left classification, total	232 (20.4)
CEAP—right C1/left C2	133 (11.7)
CEAP—right C2/left C1	99 (8.7)

*CEAP*, Clinical-Etiological-Anatomical-Pathophysiological classification; *SD*, standard deviation.

Discordant = one limb C1, one limb C2. Family history data available for 1105 of 1138 women. Limb-level CEAP classification is based on 2276 limb observations; patient-level paired CEAP classification is based on 1138 women (one paired right-left classification per woman).

At the limb level, 1564 limbs (68.7%) were classified as CEAP C1 and 712 limbs (31.3%) as CEAP C2. At the patient level, paired right-left CEAP classification showed that 666 women (58.5%) had bilateral C1 classification, 240 (21.1%) had bilateral C2 classification, and 232 (20.4%) had discordant classification. Among women with discordant classification, 133 (11.7%) had C1 in the right limb and C2 in the left limb, whereas 99 (8.7%) had C2 in the right limb and C1 in the left limb. Accordingly, C2 classification was observed in 339 right limbs (29.8%) and 373 left limbs (32.8%), corresponding to a 3.0 percentage-point higher prevalence in the left limb. This left-sided predominance was statistically significant in a paired analysis using McNemar test (matched-pairs ORs, 1.34; 95% CI, 1.04-1.74; *P* = .026; Table I).

### REXI distribution at the limb level

The overall REXI distribution at the limb level is shown in Table II. Most limbs had no saphenous reflux, with 1505 of 2276 limbs (66.1%) classified as REXI-0. REXI-1 was observed in 494 limbs (21.7%), REXI-2 in 241 limbs (10.6%), and extensive reflux, defined as REXI-3 (≥6 zones), was uncommon, occurring in 36 limbs (1.6%).

**Table II:** Limb-level distribution of Reflux Extension Index (*REXI*) categories by Clinical-Etiological-Anatomical-Pathophysiological classification (CEAP) class (n = 2276 limbs)

REXI categories	NZR range	n (% of total)	C1 limbs n (row %)	C2 limbs n (row %)
REXI-0	0	1505 (66.1)	1273 (84.6)	232 (15.4)
REXI-1	1-2	494 (21.7)	217 (43.9)	277 (56.1)
REXI-2	3-5	241 (10.6)	70 (29.0)	171 (71.0)
REXI-3	≥6	36 (1.6)	4 (11.1)	32 (88.9)
Total	–	2276 (100.0)	1564 (68.7)	712 (31.3)

*NZR*, Number of zones with reflux.

Row percentages indicate the prevalence of C1 or C2 within each REXI category. CEAP classification is limb-specific.

### Association between REXI and CEAP—limb level

At the limb level, the prevalence of CEAP C2 classification increased progressively across REXI categories, from 15.4% in REXI-0 to 56.1% in REXI-1, 71.0% in REXI-2, and 88.9% in REXI-3. This monotonic pattern was confirmed within the GEE framework, with each one-category increase in REXI associated with higher odds of CEAP C2 classification (OR for trend, 3.59; 95% CI, 3.10-4.16; *P* < .001). In population-averaged logistic regression estimated via GEEs, accounting for correlation between right and left limbs within the same participant, higher REXI categories were strongly associated with CEAP C2 classification (Table III). Compared with REXI-0, the odds of CEAP C2 classification were higher for REXI-1 (OR, 5.82; 95% CI, 4.66-7.27), REXI-2 (OR, 9.58; 95% CI, 7.20-12.73), and REXI-3 (OR, 23.18; 95% CI, 11.96-44.93), with all associations statistically significant (*P* < .001). Model discrimination was assessed using the AUROC, calculated from the predicted probabilities generated by the fitted population-averaged logistic regression model. The model yielded an AUROC of 0.755 (95% CI, 0.735-0.775), indicating acceptable discrimination. Further model details are provided in the Supplement (online only).

**Table III:** Limb-level odds ratios for Clinical-Etiological-Anatomical-Pathophysiological (CEAP) C2 classification according to Reflux Extension Index (*REXI*) category, estimated using population-averaged logistic regression with generalized estimating equations (GEEs; n = 2276 limbs from 1138 women)

REXI categories	Odds ratio	95% CI	*P* value
REXI-0	1.00	Reference	—
REXI-1	5.82	4.66-7.27	<.001
REXI-2	9.58	7.20-12.73	<.001
REXI-3	23.18	11.96-44.93	<.001
Trend per one-category increase in REXI	3.59	3.10-4.16	<.001

*CI*, Confidence interval.

Odds ratios were estimated using population-averaged logistic regression with an exchangeable working correlation structure and robust standard errors clustered at the patient level to account for correlation between right and left limbs from the same woman. CEAP C2 was the outcome (CEAP C1 the reference), and REXI-0 the reference exposure category. The trend estimate was derived by modeling REXI as an ordinal variable within the same GEE framework.

### Concordant subgroups—demographic and clinical comparison

At the patient level, women with bilateral C2 disease were significantly older than women with bilateral C1 disease (47.3 ± 15.0 vs 42.2 ± 12.6 years; mean difference, 5.2 years; 95% CI, 3.2-7.1; *P* < .001). Age category was also associated with CEAP classification (Pearson *χ*^2^ = 25.1; df = 4; *P* < .001), mainly reflecting a higher proportion of women aged ≥60 years in the bilateral C2 group than bilateral C1 group (23.3% vs 11.1%). The prevalence of venous symptoms was similar between groups (74.0% vs 73.8%; *P* = .934). Family history of varicose veins was more frequent among women with bilateral C2 disease than among those with bilateral C1 disease (86.0% vs 80.0%; Pearson *χ*^2^ = 4.08; *P* = .043).

### REXI distribution at the patient level

Table IV shows the patient-level distribution of REXI categories among women with concordant bilateral CEAP classification. Among women with bilateral C1 disease, 71.8% had REXI-0, indicating no saphenous reflux in either limb, 89.8% had REXI-0 or REXI-1, 8.4% had REXI-2 and 1.8% had REXI-3. In contrast, among women with bilateral C2 disease, only 17.5% had REXI-0, whereas 24.6% had REXI-1 and 57.9% had REXI-2 or REXI-3. The association between patient-level REXI category and bilateral CEAP classification was statistically significant (Pearson *χ*^2^ = 282.8; df = 3; *P* < .001), with a positive rank correlation between increasing REXI category and bilateral C2 classification (Spearman ρ = 0.54).

**Table IV:** Patient-level Reflux Extension Index (*REXI*) distribution in concordant subgroups (n = 906)

REXI categories	Bilateral C1 n (col %)	Bilateral C2 n (col %)	Total
REXI-0	478 (71.8)	42 (17.5)	520 (57.4)
REXI-1	120 (18.0)	59 (24.6)	179 (19.8)
REXI-2	56 (8.4)	82 (34.2)	138 (15.2)
REXI-3	12 (1.8)	57 (23.8)	69 (7.6)
Total	666 (100)	240 (100)	906 (100)

Bilateral C1 = both limbs CEAP C1; Bilateral C2 = both limbs CEAP C2.

Column percentages are within-group proportions. Spearman ρ = +0.54 (*P* < .001).

### Patient-level logistic regression—crude and adjusted

Patient-level binary logistic regression among women with concordant bilateral CEAP classification (n = 906) confirmed that the REXI category was strongly associated with bilateral C2 classification. In the crude model, compared with REXI-0, the ORs for bilateral C2 classification were 5.60 (95% CI, 3.59-8.72), 16.66 (95% CI, 10.48-26.49), and 54.06 (95% CI, 26.90-108.63) for REXI-1, REXI-2, and REXI-3, respectively (all *P* < .001; McFadden pseudo-R^2^ = 0.266). After adjustment for age and family history among women with complete data (n = 885), the corresponding associations were essentially preserved: adjusted ORs were 5.26 (95% CI, 3.34-8.29), 14.93 (95% CI, 9.31-23.95), and 51.97 (95% CI, 25.53-105.81), respectively, (all *P* < .001; McFadden pseudo-R^2^ = 0.280). Age was independently associated with bilateral C2 classification (adjusted OR per year, 1.03; 95% CI, 1.01-1.04; *P* < .001), as was family history of varicose veins (adjusted OR, 1.68; 95% CI, 1.03-2.76; *P* = .039). Adjusted ORs are shown in Table V.

**Table V:** Adjusted patient-level odds ratios (*ORs*) for bilateral C2 classification [reference: Reflux Extension Index-0 (*REXI-0*); adjusted for age and family history; n = 885 women]

Predictors	Adjusted OR	95% CI	*P* value
REXI categories	–	–	–
REXI-1 vs REXI-0	5.26	3.34-8.29	<.001
REXI-2 vs REXI-0	14.93	9.31-23.95	<.001
REXI-3 vs REXI-0	51.97	25.53-105.81	<.001
Age, per year	1.03	1.01-1.04	<.001
Family history, yes vs no	1.68	1.03-2.76	.039

*CI*, Confidence interval; *OR*, odds ratio; *REXI*, Reflux Extension Index.

Multivariable binary logistic regression; 885 women with complete data on all covariates. McFadden pseudo-R^2^ = 0.280. Area under the receiver-operating characteristic curve (AUROC) of the adjusted model: 0.837 (95% CI, 0.805-0.868).

### Patient-level discrimination—AUROC

Patient-level discrimination, assessed by AUROC in the 906 concordant women, was good. The crude model (REXI alone) achieved an AUROC of 0.815 (95% CI, 0.783-0.847). Adjustment for age and family history improved discrimination to AUROC 0.837 (95% CI, 0.805-0.868), a statistically significant gain (ΔAUROC, +0.022; *χ*^2^ = 10.68; *P* = .001).

### Adjusted predicted probabilities across REXI categories

Adjusted predicted probabilities of bilateral C2 classification by REXI category, derived by marginal-effects estimation from the multivariable model, were 8.7% (REXI-0; 95% CI, 6.2-11.1), 32.3% (REXI-1; 25.5-39.2), 56.6% (REXI-2; 48.3-64.9), and 81.3% (REXI-3; 72.0-90.7). The probability of bilateral C2 classification crossed the 50% threshold between REXI-1 and REXI-2. The increase in probability was near-uniform across categories—approximately 24 percentage-points per step (23.6, 24.3, and 24.7 points from REXI-0 to REXI-3)—with no single dominant transition (Fig 2).

**Fig 2:**
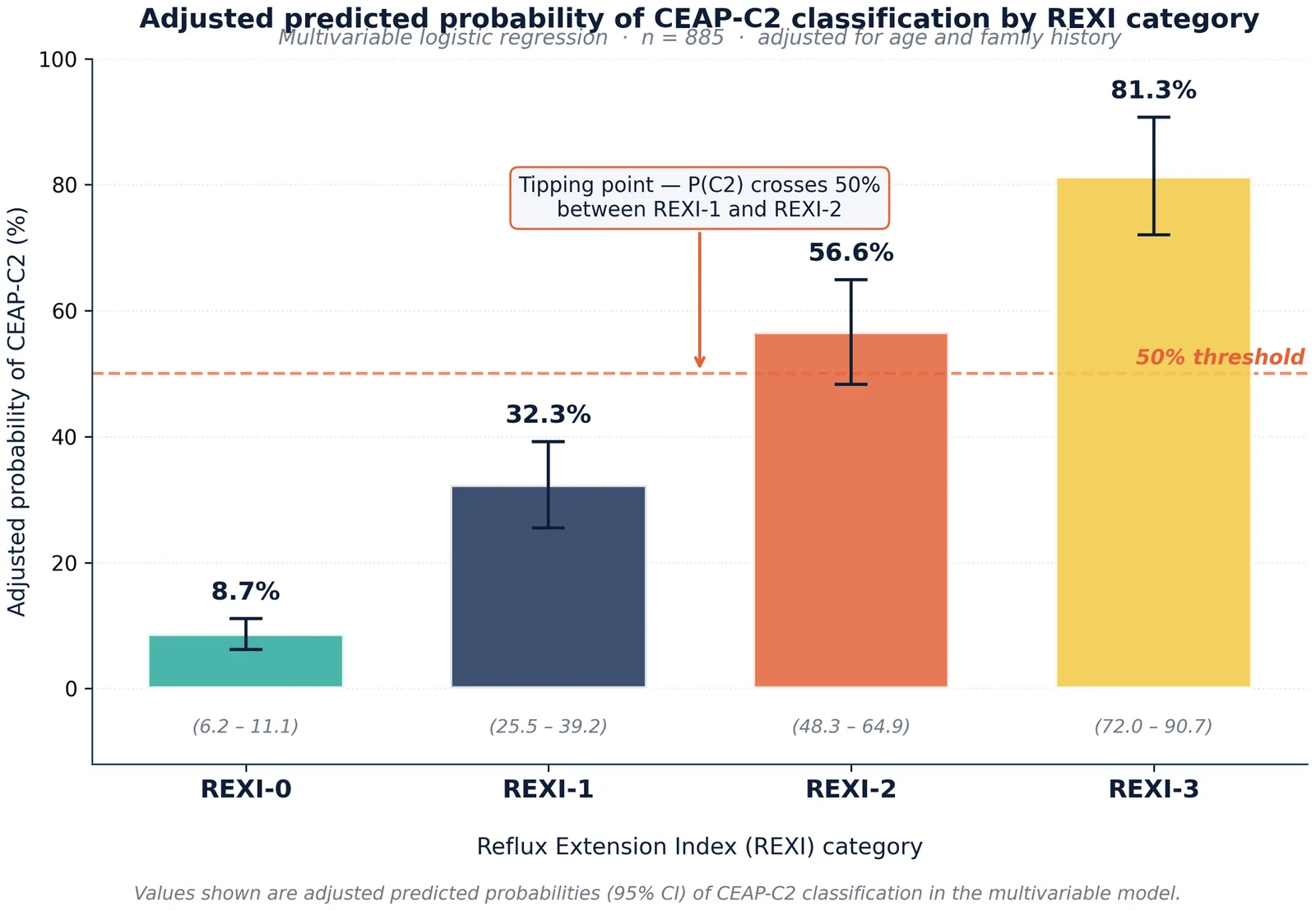
Adjusted predicted probability of Clinical-Etiological-Anatomical-Pathophysiological (*CEAP*) C2 classification by Reflux Extension Index (*REXI*) category—patient-level multivariable model (n = 885; adjusted for age and family history). Predicted probabilities [95% confidence interval (CI), REXI-0 8.7% (6.2-11.1); REXI-1 32.3% (25.5-39.2); REXI-2 56.6% (48.3-64.9); REXI-3 81.3% (72.0-90.7)]. The 50% probability threshold is crossed between REXI-1 and REXI-2—the clinical tipping point at which saphenous involvement becomes the dominant mechanism.

### Identification of bilateral C2 women

Among the 240 women with bilateral C2 disease, 42 (17.5%) had REXI-0, indicating no detectable saphenous reflux in either limb. An additional 59 women (24.6%) had REXI-1, indicating minimal saphenous reflux involving one to two zones. Therefore, 101 women (42.1%) were defined as absent or minimal saphenous reflux (REXI-0 or REXI-1). Conversely, 139 women (57.9%) had REXI-2 or REXI-3, indicating a higher saphenous reflux burden involving three or more zones. These mutually exclusive strata are summarized in Table VI.

**Table VI:** Bilateral C2 women stratified by Reflux Extension Index (*REXI*) profile (n = 240 women)

REXI profile	n	% of bilateral C2 women	Clinical interpretation
REXI-0	42	17.5	No detectable saphenous reflux; strict C1-like reflux profile
REXI-1	59	24.6	Minimal saphenous reflux involving 1-2 zones; together with REXI-0, defines the broad C1-like reflux profile
REXI-2 + REXI-3	139	57.9	Higher saphenous reflux burden involving ≥3 zones; profile more consistent with conventional reflux-associated C2 disease

The broad C1-like reflux profile was defined as REXI-0 or REXI-1. C1-like profile: saphenous reflux burden within the range observed in ≥89.8% of bilateral C1 women.

## Discussion

This is a cross-sectional analysis of saphenous reflux patterns in women with early CVD, and it introduces REXI as a quantitative framework that stratifies C1 and C2 by reflux burden. Some findings stand out. First, at the limb level, two-thirds of limbs had no detectable saphenous reflux. Second, at the patient level REXI discriminated C1 from C2 with good accuracy, alone (AUROC, 0.815) and after adjustment for age and family history (AUROC, 0.837), with the adjusted probability of C2 crossing 50% between REXI-1 and REXI-2. Third, 42.1% of bilateral C2 women presented low or no reflux.

Finally, 66.1% of all 2276 limbs were REXI-0 fitting the aesthetic, early-stage of our sample. Labropoulos et al^18^ showed that early reflux does not consistently originate at the saphenofemoral junction but may begin distally or segmentally and our findings are in line with their assumptions.

The monotonic dose-response gradient in C2 prevalence at the limb level (15.4% → 56.1% → 71.0% → 88.9%) constitutes an argument for the biological plausibility of the index. At the patient level, the adjusted probability of C2 crosses the 50% threshold between REXI-1 and REXI-2, identifying a presumable quantitative marker for early CVD.

REXI alone carries most of the discriminatory signal (patient-level AUROC, 0.815), with age and family history refining rather than replacing it (adjusted AUROC, 0.837; Δ +0.022; *P* = .001). The contrast with the limb-level AUROC of 0.755 is informative: clustering of limbs within women dilutes the signal at the limb unit, whereas at the patient REXI performs better.

Our findings align with Tan et al^12^ 2024 systematic review, which confirmed that reflux patterns predict clinical class and named the unmet need to quantify reflux degree. The junctional reflux on the venous severity score study (352 patients, 10 centers) adds support: saphenofemoral junction reflux did not predict the Venous Clinical Severity Score, confirming that no single landmark—neither junctional reflux nor maximum GSV diameter^19^—captures severity.^13^ Each landmark can nonetheless aid clinical judgment, as Navarro et al^20^ showed for GSV diameter, which correlates with reflux severity and CEAP class and helps identify limbs unlikely to need intervention. With the four-type classification of Yılmaz et al^11^ and the six-pattern taxonomy of Engelhorn et al,^7^ the literature has converged on anatomical extent—no single-point assessment—as a key determinant. REXI advances this by replacing qualitative pattern recognition with a reproducible bedside count.

The potentially useful finding is the identification, at the patient level, of a substantial subset of bilateral C2 women with a low reflux profile. Under the strict definition, 42 (17.5%) had no detectable saphenous reflux despite bilateral C2 classification; under the broad definition, 101 (42.1%) had absent or minimal reflux (0-2 zones/limb). These women constitute a population to be followed up in longitudinal trials to improve the understanding of the disease progression in the early stages of CVD.

The natural history of CVD is characterized by slow, gradual progression measured in years to decades; in the population-based Edinburgh Vein Study, only about one third of limbs with varicose veins progressed to skin changes over 13 years, at roughly 4% per year.^21^ The measurement of reflux extension at the saphenous trunks could help to monitor patients initially treated with transcutaneous methods and saphenous vein-sparing strategies to be reassigned to saphenous ablation at any subsequent visit if clinical progression, worsening symptoms, or new hemodynamic findings emerge. The conservative approach is fully reversible; ablation is permanent. This asymmetry runs in favor of watchful initial conservative management.

Equally important, current indications for saphenous ablation in C2 are largely consensus-based rather than derived from outcomes-stratified trials. The 0.5-second threshold for pathological reflux was set by expert consensus,^14^ not by trials comparing ablation with conservative management across reflux burdens. Landmark trials—Endovenous Radiofrequency Obliteration (Closure) versus Ligation and Stripping,^22^ Comparison of LAser, Surgery and foam Sclerotherapy,^23^ and the National Institute for Health and Care Excellence-commissioned studies^24^—compared ablation with surgery or foam sclerotherapy in patients with confirmed truncal reflux, but none stratified by reflux extent or distribution. The National Institute for Health and Care Excellence guidance further reinforced the need to quantify the degree of superficial venous reflux to adequately reflect disease severity.^24^ REXI does not contradict any recommendation, on the contrary, provides additional information to the CEAP, identifying patients with C1 disease significant amount of reflux and patients with C2 disease low or no reflux.

The left-sided predominance of C2 disease (McNemar OR, 1.34; 95% CI, 1.04-1.74; *P* = .026) is anatomically plausible however, the iliac segment was not assessed in our study.^25^ Our data confirm this asymmetry in a large, systematically characterized and suggest that any future REXI-based treatment algorithm should account for laterality as a secondary risk modifier.

The REXI framework also addresses the longstanding methodological gap regarding qualitative reflux descriptors. Terms such as “segmental,” “multisegmental,” or “diffuse” reflux carry no standardized quantitative meaning across studies.^6^, ^9^ REXI suggests the replacement of these with a simple, reproducible zone count that can be performed immediately at the bedside, without recourse to complex anatomical diagrams.

### Limitations

Several limitations apply. The cross-sectional design precludes causal inference and longitudinal assessment of progression. The private-clinic population had predominantly aesthetic complaints, limiting generalizability to higher-burden or public sector settings; REXI thresholds were derived from the study data, risking optimism bias; external validation is essential before clinical use. A single-operator precluded inter-rater reliability assessment of the 12-zone framework—a multioperator reproducibility study is a priority. Family history was self-reported, with 2.9% missingness (33 of 1138 women), and should be interpreted cautiously. Identifying women with C2 disease and low or absent reflux may warrant monitoring clinical progression and prospective re-evaluation in future studies. Finally, the framework quantifies only the saphenous territory; pathological perforator veins—a contested, weak-evidence target^15^—were recorded but excluded from the NZR and warrant future extension.

## Conclusions

The REXI is a simple, quantitative, and clinically applicable ordinal framework for measuring saphenous reflux extension in women with early chronic venous disease. It demonstrates a strong monotonic dose-response association with CEAP class and moderate-to-good discriminative power between C1 and C2 disease at the limb and patient level, respectively. It identifies a substantial subgroup of women clinically classified as bilateral C2 whose saphenous reflux burden is absent or minimal. These women may therefore be candidates for clinical vigilance and prospective longitudinal trials. By joining clinical classification with a potentially treatment-actionable quantitative framework, REXI may offers a rational, evidence-grounded basis for individualizing treatment decisions. Prospective multicenter validation and interventional outcome studies comparing patients with low-REXI vs high-REXI C2 disease are the required next steps.

## Author Contributions

Conception and design: CS, FM, RF

Analysis and interpretation: CS, AS

Data collection: CS

Writing the article: CS

Critical revision of the article: FM, AS, RF

Final approval of the article: CS, FM, AS, RF

Statistical analysis: AS

Obtained funding: Not applicable

Overall responsibility: FM

## Ethics approval

This study was approved by the Research Ethics Committee of the Federal University of São Paulo [Universidade Federal de São Paulo (UNIFESP); Approval No. 7.390.422]. The requirement for individual written informed consent was waived because the study used anonymized secondary data.

## Data availability

Anonymized data underlying this study are available from the corresponding author on reasonable request, subject to approval by the Universidade Federal de São Paulo (UNIFESP) Ethics Committee.

## Declaration of generative AI and AI-assisted technologies in the writing process

During the preparation of this work, the authors used Claude AI (Anthropic) to assist with concept refinement and language editing. After using this tool, the authors reviewed and edited the content as needed and take full responsibility for the content of the publication. All scientific content, conclusions, and final wording remain the sole responsibility of the human authors.

## Reporting guideline

The Strengthening the Reporting of Observational Studies in Epidemiology (STROBE) checklist for cross-sectional studies was followed. The completed checklist is provided as a Supplementary file (online only).

## Funding

None.

## Disclosures

None.

## Appendix (online only)

Supplementary Material

STROBE checklist
