# Supplementary material for: Reflux Extension Index as a novel ordinal tool for quantifying saphenous reflux burden in women with Clinical-Etiological-Anatomical-Pathophysiological classes C1 to C2 chronic venous disease
Source: J Vasc Surg Venous Lymphat Disord. 2026 Oct 10;15(1):102640. doi: 10.1016/j.jvsv.2026.102640 (PMC13769324; doi:10.1016/j.jvsv.2026.102640)
Supplement: Supplementary file 1 [file mmc1.docx]

**Supplemental Material**

# Detailed Statistical Methods

## 1 Software

Analyses were performed in **STATA/MP 18.0** (StataCorp LLC, College Station, TX, USA) and **R 4.3** (R Core Team). Receiver operating characteristic (ROC) analysis and DeLong comparisons used the pROC package in R. All tests were two-sided with α = 0.05.

## 2 Descriptive statistics

Continuous variables are reported as mean ± standard deviation (SD); categorical variables as frequencies and percentages. Group comparisons used the two-sample t-test for continuous variables and Pearson's chi-squared test (or Fisher's exact test for small expected counts) for categorical variables. Rank-order association between REXI category and CEAP class was quantified by Spearman's ρ.

## 3 Limb-level analysis — population-averaged logistic regression (generalized estimating equations)

Because each woman contributed two inherently correlated limbs, limb-level associations between REXI category and CEAP C2 classification were estimated using population-averaged logistic regression fitted by generalized estimating equations (GEE), with an exchangeable working correlation structure and robust (sandwich) standard errors clustered at the woman level to account for the correlation between right and left limbs. The outcome was CEAP class at the limb level (0 = C1, 1 = C2) and the predictor was REXI category (four levels; REXI-0 as reference), yielding population-averaged odds ratios with 95% confidence intervals for REXI-1, REXI-2, and REXI-3 versus REXI-0. Model discrimination at the limb level was assessed by the AUROC computed from the predicted probabilities of the fitted GEE model (Section 6.10).

## 4 Monotonic trend across REXI categories

The monotonic trend in CEAP C2 classification across the ordered REXI categories (REXI-0 < REXI-1 < REXI-2 < REXI-3) was assessed within the same GEE framework by modelling REXI as a single ordinal variable, yielding an odds ratio for trend per one-category increase (OR 3.59; 95% CI 3.10–4.16; p < 0.001).

## 5 Paired right–left comparison — McNemar's test

The symmetry of bilateral CEAP classification (right- versus left-limb C2) was assessed by McNemar's test for paired proportions, yielding a matched-pairs (McNemar) odds ratio and 95% CI; an odds ratio > 1 indicates left-sided predominance of C2 classification.

## 6 Patient-level analysis — binary logistic regression

6.1 Patient-level analyses were restricted to women with **concordant** bilateral CEAP classification (bilateral C1, n = 666; bilateral C2, n = 240; total n = 906). Women with discordant classification (n = 232) were described separately and excluded from the regression models, because a single bilateral outcome class could not be unambiguously assigned. Two models were fitted:

- **Crude model** — patient-level REXI category as the sole predictor of bilateral C2 classification (REXI-0 as reference), fitted on all 906 concordant women.
- **Adjusted model** — REXI category plus age (continuous, per year) and family history of varicose veins (binary), both selected a priori as biologically plausible confounders. Fitted as a complete-case analysis on the 885 women with complete covariate data (21 concordant women had missing family history).

Model fit was assessed by the McFadden pseudo-R².

6.2 **Discordant women** as a separate outcome category

**Table S.** Sensitivity analysis including discordant women as a separate outcome category: adjusted multinomial logistic regression.

| **Outcome category** | **Predictor** | **Adjusted RRR** | **95% CI** | **p-value** |
| --- | --- | --- | --- | --- |
| **Bilateral C2 vs bilateral C1** | REXI-0 | 1.00 | Reference | — |
|  | REXI-1 | 5.43 | 3.45–8.56 | <0.001 |
|  | REXI-2 | 15.53 | 9.68–24.91 | <0.001 |
|  | REXI-3 | 56.30 | 27.55–115.03 | <0.001 |
| **Discordant vs bilateral C1** | REXI-0 | 1.00 | Reference | — |
|  | REXI-1 | 5.10 | 3.47–7.51 | <0.001 |
|  | REXI-2 | 7.06 | 4.48–11.14 | <0.001 |
|  | REXI-3 | 10.05 | 4.51–22.35 | <0.001 |

RRR, relative risk ratio; CI, confidence interval; CEAP, Clinical-Etiological-Anatomical-Pathophysiological; REXI, Reflux Extension Index. Multinomial logistic regression was performed with bilateral C1 as the reference outcome category and adjusted for age as a continuous variable and family history of varicose veins. Women with complete data on all covariates were included (n = 1,105).

As a sensitivity analysis, women with discordant right–left CEAP classification were retained as a separate outcome category in multinomial logistic regression, with bilateral C1 as the reference group. After adjustment for age as a continuous variable and family history of varicose veins, increasing REXI categories were associated with both bilateral C2 and discordant classification. The gradient was substantially steeper for bilateral C2 than for discordant classification, supporting the interpretation of discordant cases as an intermediate phenotype rather than as missing or excluded observations.

## 7 Discrimination — AUROC and DeLong comparison

For each patient-level model, discrimination was quantified by the AUROC computed from the model's predicted probabilities. The crude and adjusted AUROCs were compared using DeLong's test for two correlated ROC curves, which provided the significance of the improvement in discrimination after adjustment (Δ AUROC).

## 8 Adjusted predicted probabilities — marginal effects

Adjusted predicted probabilities of bilateral C2 classification by REXI category were estimated from the adjusted model by the marginal-effects (predictive-margins) method: for each REXI category the predicted probability was computed for every woman at her observed covariate values and averaged across the 885 women, so that the estimates reflect the observed distribution of age and family history. Confidence intervals were obtained by the delta method. These predicted probabilities are reported in the main text (§3.8) and displayed in Figure 2.

## 9 Missing data

Missing covariate data were confined to the family-history variable among the concordant subgroup (21 of 906 women). The crude model used all 906 women; the adjusted model was a complete-case analysis (n = 885). No other covariate had missing data in the patient-level models.

## 10 Limb-level discrimination and sensitivity analysis (cf. §3.3)

The limb-level ROC analysis, based on predictions from the population-averaged GEE model (2,276 limbs from 1,138 women), yielded an AUROC of 0.755 (95% CI, 0.735–0.775), indicating acceptable discrimination. Overall predictive accuracy was additionally quantified by the Brier score — the mean squared difference between observed CEAP C2 status and the model's predicted probabilities — which was 0.164, lower than the null-model Brier score of 0.215 (based on the overall CEAP C2 prevalence of 31.3%), supporting improved prediction relative to a prevalence-only model. As a sensitivity analysis, model discrimination was reassessed using the continuous NZR count that underlies the REXI categorization: predicted probabilities were generated from a population-averaged GEE model with continuous NZR as the predictor, giving an AUROC of 0.757 (95% CI, 0.737–0.778) — essentially identical to that obtained with the categorized REXI (0.755). This confirms that the ordinal categorization preserved virtually all of the discriminatory information of the underlying count. Both ROC curves are shown in Figure S1.

**(A) Categorized REXI model**

**(B) Continuous NZR (sensitivity analysis)**

Figure S1. Receiver operating characteristic (ROC) curves for CEAP C2 classification at the limb level, based on predicted probabilities from population-averaged (GEE) logistic models. (A) Model with the categorized REXI variable: AUROC 0.755 (95% CI 0.735–0.775). (B) Sensitivity analysis using the continuous Number of Zones with Reflux (NZR): AUROC 0.757 (95% CI 0.737–0.778). The near-identical curves indicate that the ordinal REXI categorization preserved virtually all of the discriminatory information of the underlying count. AUROC, area under the receiver operating characteristic curve; GEE, generalized estimating equations; NZR, Number of Zones with Reflux; REXI, Reflux Extension Index.
