# Supplementary material for: Reflux Extension Index as a novel ordinal tool for quantifying saphenous reflux burden in women with Clinical-Etiological-Anatomical-Pathophysiological classes C1 to C2 chronic venous disease
Source: J Vasc Surg Venous Lymphat Disord. 2026 Oct 10;15(1):102640. doi: 10.1016/j.jvsv.2026.102640 (PMC13769324; doi:10.1016/j.jvsv.2026.102640)
Supplement: Supplementary file 2 [file mmc2.docx]

**STROBE Statement — Checklist of Items That Should Be Included in Reports of Cross-Sectional Studies**

**Manuscript title:** Reflux Extension Index (REXI) a novel ordinal tool for quantifying saphenous reflux burden in women with CEAP C1–C2 chronic venous disease

Reference: von Elm E, Altman DG, Egger M, et al. The Strengthening the Reporting of Observational Studies in Epidemiology (STROBE) statement: guidelines for reporting observational studies. Lancet. 2007;370(9596):1453–1457.

** Indicates items specifically modified for cross-sectional studies. NA = Not applicable. NZR = Number of Zones with Reflux. REXI = Reflux Extension Index. CEAP = Clinical–Etiological–Anatomical–Pathophysiological classification. GEE = generalized estimating equations. AUROC = area under the receiver operating characteristic curve.*

| **Item** | **Recommendation** | **Reported** | **Location in Manuscript / Supplement** |
| --- | --- | --- | --- |
| **TITLE AND ABSTRACT** | | | |
| 1a | Indicate the study's design with a commonly used term in the title or the abstract | Yes | Title; Abstract — Methods paragraph (“Cross-sectional study of women…”) |
| 1b | Provide in the abstract an informative and balanced summary of what was done and what was found | Yes | Abstract (Objective, Methods, Results, Conclusions) |
| **INTRODUCTION** | | | |
| 2 | Explain the scientific background and rationale for the investigation being reported | Yes | Section 1 (Introduction) |
| 3 | State specific objectives, including any pre-specified hypotheses | Yes | Abstract — Objective; Section 1 (final paragraph) |
| **METHODS** | | | |
| 4 | Present key elements of study design early in the paper | Yes | Section 2.1 (Study Design and Setting) |
| 5 | Describe the setting, locations, and relevant dates, including periods of recruitment, exposure, follow-up, and data collection | Yes | Section 2.1 — Nova Lima, Brazil; 1 January 2019 – 31 July 2023 |
| 6 | Give the eligibility criteria, and the sources and methods of selection of participants | Yes | Section 2.2 (Study Population); full exclusion criteria in Supplement Section 2 |
| 7 | Clearly define all outcomes, exposures, predictors, potential confounders, and effect modifiers. Give diagnostic criteria, if applicable | Yes | Sections 2.3 (CEAP), 2.4 (Duplex), 2.5 (12-zone framework and NZR), 2.6 (REXI), 2.7 (Concordant subgroups) |
| 8* | For each variable of interest, give sources of data and details of methods of assessment (measurement). Describe comparability of assessment methods if there is more than one group | Yes | Section 2.4; single trained operator; equipment specified; Supplement Section 4 (acquisition protocol) |
| 9 | Describe any efforts to address potential sources of bias | Yes | Sections 2.1 (ethics, anonymised data), 2.2 (consecutive recruitment, pre-specified exclusions), 4.1 (Limitations) |
| 10 | Explain how the study size was arrived at | Yes | Section 2.2 — 1,720 consecutive women screened; 1,138 met inclusion criteria (all eligible included) |
| 11 | Explain how quantitative variables were handled in the analyses. If applicable, describe which groupings were chosen and why | Yes | Section 2.6 (REXI thresholds: 0, 1–2, 3–5, ≥ 6); Section 2.8 |
| 12a | Describe all statistical methods, including those used to control for confounding | Yes | Section 2.8; Supplement Section 6 — limb-level population-averaged logistic regression (GEE); patient-level crude and adjusted logistic regression |
| 12b | Describe any methods used to examine subgroups and interactions | Yes | Sections 2.7, 2.8; Supplement Section 6 |
| 12c | Explain how missing data were addressed | Yes | Section 2.8 — complete-case analysis for family history (2.9% missing, 33/1,138); Section 4.1; Table 5 footnote |
| 12d* | If applicable, describe analytical methods taking account of sampling strategy | NA | N/A — consecutive sampling; no complex probability sampling |
| 12e | Describe any sensitivity analyses | Yes | Supplement Section 6.10 — ordinal REXI vs continuous NZR discrimination; Brier score |
| **RESULTS** | | | |
| 13a* | Report numbers of individuals at each stage of study | Yes | Section 2.2 (1,720 screened → 1,138 included → 906 concordant → 885 complete cases); Supplement Section 1 (Table S0) |
| 13b* | Give reasons for non-participation at each stage | Yes | Section 2.2; full list in Supplement Section 2 |
| 13c* | Consider use of a flow diagram | Yes | Supplement Section 1 — participant flow table (Table S0) |
| 14a* | Give characteristics of study participants and information on exposures and potential confounders | Yes | Table 1; Section 3.1; Section 3.4 |
| 14b* | Indicate number of participants with missing data for each variable of interest | Yes | Family history missing in 33/1,138 (2.9%); 21/906 in the concordant analysis set; complete-case adjusted model in 885 women (Table 1 footnote; Section 4.1) |
| 15* | Report numbers of outcome events or summary measures | Yes | Tables 2–6; Sections 3.2–3.9 |
| 16a | Give unadjusted estimates and, if applicable, confounder-adjusted estimates and their precision. Make clear which confounders were adjusted for and why | Yes | Tables 3, 5; Sections 3.3, 3.6; age and family history included as a priori confounders |
| 16b | Report category boundaries when continuous variables were categorized | Yes | Section 2.6 — REXI-0 (NZR = 0), REXI-1 (1–2), REXI-2 (3–5), REXI-3 (≥ 6) |
| 16c | If relevant, consider translating estimates of relative risk into absolute risk for a meaningful time period | NA | N/A — cross-sectional; absolute risks reported as adjusted predicted probabilities (Section 3.8, Figure 2) |
| 17 | Report other analyses done — e.g. analyses of subgroups and interactions, and sensitivity analyses | Yes | Section 3.9 (strict/broad C1-like definitions); Supplement Section 6.10 (sensitivity analyses) |
| **DISCUSSION** | | | |
| 18 | Summarise key results with reference to study objectives | Yes | Section 4 — opening paragraphs |
| 19 | Discuss limitations of the study, taking into account sources of potential bias or imprecision | Yes | Section 4.1 (Limitations) |
| 20 | Give a cautious overall interpretation of results considering objectives, limitations, multiplicity of analyses, results from similar studies, and other relevant evidence | Yes | Section 4 (Discussion); Section 5 (Conclusions) |
| 21 | Discuss the generalisability (external validity) of the study results | Yes | Section 2.2; Section 4.1 (Limitations) |
| **OTHER INFORMATION** | | | |
| 22 | Give the source of funding and the role of the funders for the present study | Yes | Statements — Funding: None (no external or commercial funding) |
